# A single question regarding mobility in the World Health Organization quality of life questionnaire predicts 3-year mortality in patients receiving chronic hemodialysis

**DOI:** 10.1038/s41598-017-12276-9

**Published:** 2017-09-20

**Authors:** Hsiu-Ho Wang, Miao-Chun Ho, Kuan-Yu Hung, Hui-Teng Cheng

**Affiliations:** 1 Department of Nursing, School of Nursing, Yuanpei University of Medical Technology, Hsin Chu City, Taiwan; 2 Department of Nursing, National Taiwan University Hospital, Hsin-Chu Branch, Hsin Chu City, Taiwan; 3 Department of Internal Medicine, National Taiwan University Hospital, Hsin-Chu Branch, Hsin Chu City, Taiwan

## Abstract

Low quality of life, depression and poor quality of sleep are associated with increased mortality in hemodialysis patients. It is not clear which factor has the highest predictive power and what the core element is to explain the predictability. We thus conducted a prospective cohort study that included 151 hemodialysis adults. Three traits of interest were assessed by World Health Organization Quality of Life questionnaire, an abbreviated version (WHOQOL-BREF), Taiwanese Depression Questionnaire, and Athens Insomnia Scale, respectively. They were followed for more than 3 years and the all-cause mortality was 30.5%. The prevalence of quality of life at the lowest tertile, depression and poor quality of sleep was 19.9%, 43.0% and 74.2%, respectively. Discriminant analysis showed the standardized coefficient of each factor as 0.813, −0.289 and 0.066, indicating the highest discriminating power by quality of life to predict mortality. Question 15 “how well are you able to get around?” in the physical health domain of WHOQOL-BREF independently associated a hazard ratio of mortality 0.623 (95% confidence interval 0.423-0.918). Subjective perception of overall quality of life was more related to psycho-social-environmental factors. In conclusion, mobility is an independent and powerful predictor to long term mortality in patients on chronic hemodialysis.

## Introduction

Quality of life is one of the major indicators for general well-beings^1^ and a key concern when evaluating the acceptability of a particular treatment^2,3^. Epidemiological studies on general population have demonstrated that people with better quality of life may live longer^4,5^. Quality of life is also proved to be a reliable predictor for short term and long term mortality in many pathological conditions, including certain types of malignancy^6,7^ and diseases such as atrial fibrillation^8^, pulmonary arterial hypertension^9^ and chronic kidney disease^10^.

For patients who enter end stage renal disease and undergo maintenance hemodialysis, removal of uremic toxin and more liberty in water and food intake may improve their quality of life whereas frequent visit to dialysis facilities, needle punctures and progression of the underlying disease and the associated complications such as cardiovascular diseases may cause the opposite. Two recent large-scale studies show that around half of hemodialysis patients experience no change in quality of life, while a quarter reports an improvement and an equal number finds a decline^11,12^. One of the psychological factors that is strongly associated with low quality of life is depression^13^. Meanwhile, a majority of depressive hemodialysis patients also suffers poor quality of sleep^14^. Poor quality of sleep itself has substantial negative effect on quality of life both in general population^15^ and patients on hemodialysis^16^.

The prevalence of depression in hemodialysis patients is remarkably high, ranging from 5% to 35% and up to 75%^7,14,17^ compared to that in general population (~1.3%)^15^. The prevalence of poor quality of sleep in this group may be even higher, ranging from 53.3% to 71%^16,18^. In other words, these three agonizing conditions, namely low quality of life, depression and poor quality of sleep, are very commonly seen and related to one another. Each one of them on its own is associated with higher mortality in hemodialysis patients compared to those without^7,18,19^. On the other hand, each of these three conditions is associated with distinctive biological, psychological and social factors. Whether they may have differential power to influence survival is an intriguing question. Besides there has been no study focusing on whether they are actually independent factors to predict mortality.

To answer these two important questions, we conducted a prospective study to follow the survival of a group of hemodialysis patients for more than 3 years. Our attempt was to build a model that includes all these three indicators to examine whether they are independently associated with mortality. In addition, we also compared the diagnostic ability of these three indicators. No such analysis has been done in patients with other diseases either. To assess the quality of life we used the World Health Organization Quality of Life assessment questionnaire, an abbreviated version (WHOQOL-BREF). This instrument allowed us to investigate the predictability of various aspects in quality of life to mortality including physical, psychological, social and environmental factors.

## Results

### Low quality of life, depression and poor quality of sleep are individually associated to 3-year mortality in patients receiving chronic hemodialysis

The mortality was 46, or 30.5%, in 3-plus years. The non-survivors had higher average scores indicating depression, dismal qualities of life (within the lowest tertile) and sleep. Significantly more non-survivors experienced poor quality of life and depression, but the proportions of having poor quality of sleep were similarly high in survivors versus non-survivors (p = 0.246). The non-survivors had lower serum levels of albumin and creatinine. Survivors were more likely to have received longer school education (p = 0.001), to be employed (p = 0.043) and to have higher monthly income (p = 0.071) (Table 1).

**Table 1:** Characteristics in demography and biochemistry between survivors and non-survivors. Data are presented as mean ± standard deviation, or number (percentage).

Variables	Total (n = 151)	Survivor (n = 105, 69.5%)	Non-survivor (n = 46, 30.5%)	*p* value of t-, z- or Chi square test
Quality of life score	79.87 ± 16.29	82.77 ± 15.90	73.26 ± 15.36	**0.001**
% QOL score = <65	30(19.9%)	16(15.2%)	14(30.4%)	**0.032**
Domain 1 score average	18.23 ± 5.12	19.31 ± 4.97	15.76 ± 4.61	**0.000**
Domain 2 score average	15.88 ± 4.43	16.55 ± 4.30	14.35 ± 4.38	**0.005**
Domain 3 score average	12.52 ± 2.18	12.84 ± 2.08	11.80 ± 2.26	**0.007**
Domain 4 score average	28.42 ± 5.51	29.09 ± 5.40	26.89 ± 5.52	**0.024**
Depression TDQ score	18.86 ± 14.47	16.63 ± 13.98	23.96 ± 14.44	**0.004**
% TDQ score > = 19	65(43.0%)	39(37.1%)	26(56.5%)	**0.027**
Quality of sleep score	10.36 ± 6.05	9.62 ± 5.97	12.06 ± 5.96	**0.022**
% QOS score > = 6	112(74.2%)	75(71.4%)	37(80.4%)	0.246
Age	64.62 ± 13.91	62.33 ± 13.79	69.85 ± 12.88	**0.002**
Gender (woman %)		48(45.7%)	29(63.0%)	0.050
Marital status			^χ^ ^2^ = 3.06	0.382
married	118(78.1%)	82(78.1%)	36(78.3%)	
single	10(6.6%)	9(8.6%)	1(2.2%)	
widowed	19(12.6%)	12(11.4%)	7(15.2%)	
divorced	4(2.6%)	2(1.9%)	2(4.3%)	
Education			^χ^ ^2^ = 19.40	**0.001**
no	18(11.9%)	11(10.5%)	7(15.2%)	
<6 years	67(44.4%)	36(34.3%)	31(67.4%)	
6–9 years	21(13.9%)	18(17.1%)	3(6.5%)	
9–12 years	34(23.2%)	31(29.5%)	3(6.5%)	
college	11(7.3%)	9(8.6%)	2(4.3%)	
Occupation			^χ^ ^2^ = 4.08	**0.043**
unemployed	132(87.4%)	88(83.8%)	44(95.7%)	
employed	19(12.6%)	17(16.2%)	2(4.3%)	
Religion			^χ^ ^2^ = 6.30	0.278
nil	49(32.5%)	32(30.5%)	17(37.0%)	
Buddhism	65(43.0%)	49(46.7%)	16(34.8%)	
Christianity	3(2.0%)	3(2.9%)	0(0.0%)	
Catholicism	12(7.9%)	9(8.6%)	3(6.5%)	
Taoism	21(13.9%)	11(10.5%)	10(21.7%)	
miscellaneous	1(0.7%)	1(1.0%)	0(0.0%)	
Monthly income (USD)			^χ^ ^2^ = 7.02	0.071
<300	87(57.6%)	55(52.4%)	32(69.6%)	
300–1000	37(24.5%)	26(24.8%)	11(23.9%)	
1000–2000	17(11.3%)	16(15.2%)	1(2.2%)	
>2000	10(6.6%)	8(7.6%)	2(4.3%)	
Living			^χ^ ^2^ = 2.75	0.252
alone	5(3.3%)	3(2.9%)	2(4.3%)	
with family	139(92.1%)	99(94.3%)	40(87.0%)	
other	7(4.6%)	3(2.9%)	4(8.7%)	
Diabetes mellitus	84(55.6%)	58(55.2%)	26(56.5%)	0.881
BUN	75.29 ± 20.99	76.65 ± 21.58	72.20 ± 19.44	0.231
Creatinine	10.34 ± 2.58	10.70 ± 2.59	9.50 ± 2.41	**0.008**
Sodium	136.88 ± 4.13	137.10 ± 4.22	136.37 ± 3.90	0.315
Potassium	4.44 ± 0.97	4.43 ± 0.89	4.48 ± 1.12	0.773
Calcium	9.30 ± 0.87	9.37 ± 0.83	9.16 ± 0.93	0.174
Phosphate	4.90 ± 1.47	5.00 ± 1.50	4.68 ± 1.37	0.221
Ca x P product	45.70 ± 14.65	46.96 ± 15.15	42.84 ± 13.17	0.113
Albumin	3.65 ± 0.37	3.70 ± 0.36	3.52 ± 0.35	**0.004**
Glucose	153.23 ± 65.77	147.13 ± 59.25	167.13 ± 77.60	0.086
AST	18.48 ± 10.10	18.24 ± 9.65	19.02 ± 11.15	0.662
ALT	19.13 ± 20.02	19.64 ± 20.31	17.98 ± 19.50	0.641
Kt/V of urea	1.34 ± 0.22	1.34 ± 0.21	1.35 ± 0.24	0.731
Dialysis vintage	4.57 ± 3.90	4.24 ± 3.63	5.32 ± 4.40	0.118
ALKP	307.64 ± 138.57	294.71 ± 127.40	337.15 ± 158.79	0.083
Triglyceride	169.78 ± 111.93	172.65 ± 123.31	163.22 ± 80.99	0.635
Cholesterol	159.82 ± 34.87	160.87 ± 35.83	157.43 ± 32.83	0.579
Ferritin	460.78 ± 444.50	441.99 ± 264.48	503.68 ± 703.01	0.434
i-parathyroid hormone	338.04 ± 271.38	347.49 ± 283.56	316.46 ± 242.83	0.520
Hemoglobin	10.78 ± 1.51	10.80 ± 1.54	10.73 ± 1.45	0.772
MCV	90.58 ± 8.97	90.08 ± 9.81	91.72 ± 6.61	0.302
Platelet	177.07 ± 64.92	170.18 ± 59.98	192.80 ± 73.28	**0.048**

ALKP: alkaline phosphatase, ALT: Alanine aminotransferase, AST: Aspartate aminotransferase, BUN: blood urea nitrogen, Ca x P: calcium and phosphate product, i-parathyroid hormone: intact parathyroid hormone, Kt/V: dialysis adequacy by dialyzer clearance of urea (K) times time (t) divided by volume (V) of distribution of urea, MCV: mean corpuscular volume of red blood cell, QOL: quality of life, QOS: quality of sleep, USD: US dollar.

We applied Cox proportional hazard regression analysis to calculate the hazard ratio of mortality. In univariate analysis, the hazard ratio by one-point increment in quality of life was 0.966 (p < 0.001) (Supplemental Table S1). We further compared the hazard ratios of mortality among each quartile of quality of life scores (Table 2). The mortality was 5.586 times higher (p < 0.001) in those with the lowest scores and 2.174 times higher (p = 0.033) in the next group than those with the best quality of life (score 85–112; no one scored 113–140). Kaplan-Meier survival analysis showed a significant difference in mortality among these groups (log-rank test, p = 0.002, Supplemental Figure S1A).

**Table 2:** Hazard ratio of mortality in association with quality of life and its 4 domains, depression and quality of sleep by Cox proportional hazard regression model. Data are shown as hazard ratio, calculated by exponential of beta (Exp(B)), with 95% confidence interval.

**QOL score**	**HR**	**95% CI**		***p value***
28–56	5.586	2.025	15.411	**0.000**
57–84	2.174	1.062	4.448	**0.034**
85–112	Ref.			
113–140	death = 0 total = 4			
TDQ score				
0–8	Ref.			
9–16	0.928	0.330	2.606	0.887
17–54	2.476	1.178	5.205	**0.017**
Sleep score				
0–5	ref			
6–12	1.304	0.57	2.979	0.530
13–18	1.512	0.646	3.537	0.341
19–24	3.053	1.211	7.698	**0.018**
Domains of quality of life				
Domain 1 (physical health)	0.886	0.835	0.940	**0.000**
Domain 2 (psychological)	0.899	0.835	0.967	**0.004**
Domain 3 (social)	0.830	0.735	0.937	**0.003**
Domain 4 (environmental)	0.934	0.885	0.987	**0.015**
Domain 1				
7–15	9.527	1.284	70.716	**0.028**
16–25	4.381	0.592	32.440	0.148
26–35	Ref.			
Domain 2				
6–14	1.882	0.446	7.948	0.390
15–22	0.959	0.223	4.115	0.955
23–30	Ref.			
Domain 3				
4–8	13.060	3.251	52.468	**0.000**
9–14	2.774	0.990	7.775	0.052
15–20	Ref.			
Domain 4				
9–20	3.489	1.172	10.385	**0.025**
21–32	1.814	0.802	4.101	0.152
33–45	Ref.			

HR: hazard ratio; CI: confidence interval.

The average score of each domain in quality of life was associated with mortality (Table 2). In domains of physical health, social relationships and environment, the hazard ratio of mortality in the the lowest tertile group was 9.527 times, 13.060 times and 3.489 times higher than that in the highest group, respectively (p = 0.028, p < 0.001 and p = 0.025).

The hazard ratio of mortality was 1.027 (p = 0.005) by one-point increment in depression, and 1.059 (p = 0.017) by one-point increment in quality of sleep (Supplemental Table S1). The hazard ratio of mortality in those at the highest tertile of depression was 2.467 times higher than that at the lowest tertile (p = 0.017, Table 2). Kaplan-Meier analysis revealed a significant difference in mortality among them (log-rank test, p = 0.009, Supplemental Figure S1B). Those at the highest quartile of quality of sleep had the hazard ratio of mortality 3.053 times higher than that of the reference group (p = 0.018, Table 2) although the mortality among quartile groups did not reach statistical difference by Kaplan-Meier survival analysis (log-rank test, p = 0.082, Supplemental Figure S1C).

### Multivariate analysis suggests independence of quality of life from depression and quality of sleep in prediction to mortality

Quality of life remained to be associated with mortality when paired with another factor or all three factors included in the Cox model (Supplemental Table S2). In univariate Cox model (Supplemental Table S1), we found a handful of items that were associated with mortality, including age, creatinine, albumin, glucose and alkaline phosphatase. We then included age and albumin to examine their effects. Quality of life and depression, but not quality of sleep, remained significant. When both depression and quality of life were considered, either one showed significant association but age still did. When other factors including education, alkaline phosphatase and glucose were included, the effect of age on predicting mortality became insignificant.

### Discriminant analysis reveals a higher discriminating power by quality of life than depression and quality of sleep to predict mortality, and physical health has the highest predictability

To determine which of the three conditions has the highest power to predict mortality we took advantage of discriminant analysis to calculate the standardized canonical discriminant function coefficients in relation to mortality (survival or death, as a binary outcome; Table 3). Higher absolute value of standardized coefficient indicates a stronger discrimination power to the dependent variable. We first exercised the Box’s M Test of Equality of Covariance Matrices and calculated the Wilks’ Lambda value. Non-significance in the first and significance in the latter indicates appropriateness to run linear discriminate analysis. We found quality of life has a higher standardized coefficient, 0.813, than depression, −0.289. A close-to-zero coefficient, 0.066, by quality of sleep reflects its minor influence on mortality. We further performed the discriminant analysis on the 4 domains regarding quality of life. By absolute values, domain1 (physical health) had a higher coefficient than domain 3 (social relationships) and domain 4 (environmental aspect), while domain 2 (psychological aspect) had a very low coefficient.

**Table 3:** Discriminant analysis among quality of life, depression and quality of sleep and among individual domains in quality of life in prediction to mortality.

Discriminant analysis of quality of life, depression and quality of sleep in prediction to mortality				Discriminant analysis of 4 domains in quality of life in prediction to mortality		
Box’s M Test of Equality of Covariance Matrices 2.610, p = 0.864				Box’s M Test of Equality of Covariance Matrices 12.299, p = 0.295		
Wilks’ Lambda 0.925, Chi square = 11.494, df = 3, p = 0.009				Wilks’ Lambda 0.887, Chi square = 17.615, df = 4, p = 0.001		
	Standardized Canonical Discriminant Function Coefficients	Structure Matrix			Standardized Canonical Discriminant Function Coefficients	Structure Matrix
Quality of life	0.813	0.984		Domain1	1.024	0.949
Depression	−0.289	−0.0845		Domain2	−0.020	0.662
Quality of sleep	0.066	−0.667		Domain3	0.402	0.630
				Domain4	−0.404	0.524

### Logistic regression analysis, receiver operating characteristic curve and information value plot all indicate quality of life as a better predictor to mortality than depression and quality of sleep

Another way to compare the influential power of a variable to mortality is based on odds ratio that may be obtained by logistic regression. To make the odds ratios comparable among variables they should be measured by the same scale. We did the transformation as detailed in the Methods section. The odds ratio of mortality in relation to quality of life was 1.040, higher than that in relation to depression, 1.017, and quality of sleep, 1.015. The positive prediction values were 66.7%, 40% and 50%, respectively (Supplemental Table S3). Another way to illustrate the diagnostic ability is by Receiver Operating Characteristic curve (Supplemental Figure S2). Quality of life showed the highest value of area under curve (0.669). The middle and the lowest ones were depression and quality of sleep, respectively (0.654 and 0.620). However, the differences between them were statistically insignificant (see Methods for statistics used). Information value plot of the three factors, and four domains in quality of life, also illustrated the influence to mortality of quality of life and its physical health domain (Supplemental Figure S3).

### Single individual questions in the WHOQOL-BREF questionnaire display higher predictability to mortality

We next attempted to narrow down which individual questions in the quality of life questionnaire may differentiate the mortality. We first compared between survivors and non-survivors the score rank of each question by Mann-Whitney U test and the response distribution by Komolgorov-Smirnov Z test and by 5 × 2 contingency-table [5 responses by 2 groups (survivors versus non-survivors)] Chi-square test and Fisher’s exact test (Supplemental Table S4). These two groups of subjects responded differently only in a handful of questions. Questions 10, 11, 14, 15 and 18 showed significant differences in all three tests. We next calculate the weights of evidence and the information values of individual questions. Questions 10, 11, 14 and 15 had the highest information values (Supplemental Figure S3). We then run Cox proportional hazard regression model to find association to mortality for each question (Supplemental Table S5), and for sum of those key questions (Table 4). With multiple variables included, questions 10, 15 (both in domain 1 “physical health”) and 14 (in domain 4 “environmental”) still showed significant association with mortality. When all three questions are included, question 15 was more likely associated with mortality (p = 0.071). To further identify the most distinguishing one, pairs of these three questions were included in the model. When paired with either question 10 or 14, question 15, but not the other, remained significantly associated with mortality (Table 4).

**Table 4:** Hazard ratio of mortality in association with individual questions in the quality of life questionnaire (WHO-QOL-BREF) by Cox proportional hazard regression model. Data are shown as hazard ratio, calculated by exponential of beta (Exp(B)), with 95% confidence interval.

		HR	95% CI		p value
Domain 1 (physical health)					
Q10		**0.577**	**0.384**	**0.867**	**0.008**
Q15		**0.598**	**0.431**	**0.829**	**0.002**
Q18		0.822	0.585	1.154	0.257
Domain 2 (psychological)					
Q11		0.780	0.549	1.108	0.166
Domain 4 (environmental)					
Q14		**0.676**	**0.481**	**0.950**	**0.024**
Three questions (Q10, Q15, Q14) in					
	Q15	0.686	0.456	1.033	0.071
	Q10	0.699	0.450	1.085	0.110
	Q14	0.852	0.574	1.264	0.426
Two questions in					
Q15, Q10	Q15	**0.631**	**0.442**	**0.899**	**0.011**
	Q10	0.698	0.450	1.083	0.109
Q15, Q14	Q15	**0.623**	**0.423**	**0.918**	**0.017**
	Q14	0.850	0.576	1.256	0.415
Q10, Q14	Q10	**0.611**	**0.402**	**0.929**	**0.021**
	Q14	**0.705**	**0.502**	**0.991**	**0.045**
Sum of three questions					
Q10 + Q15 + Q14		**0.755**	**0.645**	**0.884**	**0.000**
Sum of five Questions					
Q10 + Q15 + Q14 + Q11 + Q18		**0.847**	**0.763**	**0.940**	**0.002**

Model includes TDQ depression score, age, gender, education, albumin, glucose and alkaline phosphatase.

HR: hazard ratio; CI: confidence interval.

Question 10: Do you have enough energy for everyday life?

Question 11: Are you able to accept your bodily appearance?

Question 14: To what extent do you have the opportunity for leisure activities?

Question 15: How well are you able to get around?

Question 18: How satisfied are you with your capacity for work?

### Subjective perception of worse quality of life does not necessarily indicate high mortality

As we identified the questions in the WHOQOL-BREF questionnaire that may predict higher mortality, it becomes interesting to know whether these questions also reflect a subjective perception of worse quality of life. The question 1 “How would you rate your quality of life?” is a yardstick to measure the responder’s perception of overall quality of life. We therefore compare the answers to other questions with that to question 1. If the answer is the same as that to question 1, that particular question may reflect the subjective perception of quality of life. We reported results from three tests, Mann-Whitney U test, Komolgorov-Smirnov Z test and 5 × 2 contingency-table Fisher’s exact test (Table 5). Regarding questions 10, 14, and 15, the answers to questions 14 and 15 from the non-survivors were different from that to question 1, but the answer to question 10 was largely similar to that to question 1. Questions 8 and 27 were answered similarly as question 1.

**Table 5:** Comparison in responses between question 1 and all other questions in the WHOQOL-BREF questionnaire by Mann-Whitney U test, Kolmogorov-Smirnov Z test and 5 × 2 contingency-table Fisher’s exact test (only p values reported).

Question pairs	Total (n = 151)			Survivor (n = 105)			Non-survivor (n = 46)		
	Mann-Whitney U test (p value)	Kolmogorov- Smirnov Z test(p value)	Fisher’s exact test (p value)	Mann-Whitney U test (p value)	Kolmogorov- Smirnov Z test(p value)	Fisher’s exact test (p value)	Mann-Whitney U test (p value)	Kolmogorov- Smirnov Z test(p value)	Fisher’s exact test (p value)
Q1_Q2	0.000	0.000	0.000	0.000	0.000	0.000	0.000	0.000	0.000
Q1_Q3	**0.104**	0.001	0.000	0.031	0.003	0.000	**0.760**	**0.490**	0.008
Q1_Q4	0.000	0.000	0.000	0.000	0.000	0.000	0.001	0.002	0.000
Q1_Q5	0.000	0.001	0.001	0.002	0.020	0.018	**0.096**	**0.051**	0.012
Q1_Q6	0.004	0.005	0.000	0.014	0.030	0.008	**0.101**	**0.087**	0.004
Q1_Q7	0.000	0.005	0.002	0.001	**0.064**	0.012	0.016	**0.144**	0.040
Q1_Q8	**0.982**	**1.000**	**0.974**	**0.870**	**1.000**	**0.994**	**0.897**	**0.995**	**0.583**
Q1_Q9	0.002	0.032	0.015	0.029	**0.234**	**0.053**	0.036	**0.227**	**0.202**
Q1_Q10	0.001	**0.141**	0.005	0.012	**0.064**	0.008	**0.775**	**0.144**	0.040
Q1_Q11	**0.814**	**0.895**	**0.265**	**0.761**	**1.000**	**0.782**	**0.876**	**0.490**	0.013
Q1_Q12	**0.055**	**0.091**	0.002	0.026	**0.175**	0.039	**0.773**	**0.342**	0.009
Q1_Q13	**0.374**	**0.535**	**0.237**	**0.454**	**0.974**	**0.841**	0.000	**0.661**	**0.052**
Q1_Q14	0.000	0.000	0.000	0.046	0.030	0.001	0.000	0.001	0.000
Q1_Q15	**0.164**	0.005	0.000	**0.417**	**0.091**	0.000	0.009	0.001	0.000
Q1_Q16	0.000	0.000	0.000	0.000	0.000	0.000	**0.118**	**0.087**	0.011
Q1_Q17	**0.175**	**0.630**	**0.160**	**0.501**	**0.995**	**0.734**	0.007	**0.661**	**0.178**
Q1_Q18	0.002	0.008	0.001	0.042	**0.175**	**0.070**	**0.093**	**0.051**	0.018
Q1_Q19	0.021	0.005	0.000	0.091	0.044	0.002	0.042	**0.227**	0.024
Q1_Q20	0.000	0.044	0.003	0.004	**0.234**	0.025	**0.734**	**0.342**	**0.206**
Q1_Q21	**0.528**	**1.000**	**0.620**	**0.565**	**0.995**	**0.502**	0.001	**1.000**	**0.957**
Q1_Q22	0.000	0.000	0.000	0.000	0.008	0.000	0.000	**0.087**	0.003
Q1_Q23	0.000	0.000	0.000	0.000	0.005	0.000	0.000	0.004	0.000
Q1_Q24	0.000	0.000	0.000	0.000	0.000	0.000	0.000	0.002	0.000
Q1_Q25	0.000	0.000	0.000	0.000	0.001	0.001	0.000	0.002	0.000
Q1_Q26	0.003	0.000	0.000	0.035	0.002	0.000	0.029	**0.144**	**0.057**
Q1_Q27	**0.341**	**0.984**	**0.554**	**0.567**	**1.000**	**0.974**	**0.376**	**0.949**	**0.264**
Q1_Q28	0.000	0.000	0.000	0.000	0.000	0.000	0.001	**0.144**	0.006

## Discussion

Our thorough investigation has led to four new and insightful conclusions. First, each of three factors, namely quality of life, depression and quality of sleep, is associated with higher mortality in patients undergoing hemodialysis (Tables 1 and 2, Supplemental Figure 1). Quality of life remained independently to be associated with mortality when all three factors are considered (Supplemental Table S2). Second, quality of life has the strongest discriminating power to predict mortality among three factors (Table 3, Supplemental Fig S3, Supplemental Table S3). The physical health domain is the one with the highest predictability (Table 3, Supplemental Figure S3). Third, a few key questions in the WHOQOL-BREF questionnaire, including questions 10, 14 and 15 (Table 4 and Supplemental Table S4), but not the total score (Supplemental Table S2), are associated with mortality independently from depression, age, gender, education, albumin, alkaline phosphatase and glucose levels. Question 15 “how well are you able to get around?” stands out as a powerful and independent one to predict mortality when paired with either question 10 “Do you have enough energy for everyday life?” or 14 “To what extent do you have the opportunity for leisure activities?” (Table 4). Fourth, mobility (question 15) and accessibility to leisure activities (question 14) are not linked to the subjective perception of quality of life in non-survivors (Table 5). To all subjects, it is the environmental safety and feeling of being respected that match the subjective perception.

It is of great interest to know which of the three factors is the best one to predict long term mortality in patients on maintenance hemodialysis as they are correlated with each other and individually are associated with increased mortality. When all three are included in the Cox proportional hazard model, only quality of life stands out to remain associated with mortality. Its prominent role in prediction is further proved by the discriminant analysis, and by the odds ratios calculated by logistic regression. The positive predictive value and the information value of quality of life is also the highest among three factors. Quality of life also displays the largest area under curve in the ROC plot although the difference between any pairs does not reach statistical significance.

The main reason that quality of life may well predict mortality is likely because it is a comprehensive measure that covers many different aspects in health. Depression is more related to mental health, and quality of sleep is one of numerous physiological indictors. With this thought in mind, we found it very interesting to rank the contributions of different domains in quality of life. Studies have demonstrated the association with mortality of physical function and mental health in patients on hemodialysis^12,20^ or with heart failure^21^ but they have not compared the predictive power between these two factors. Our result shows that physical health is the most important component in terms of prediction of future survival.

One intriguing finding in our analysis is that the total score of quality of life becomes not associated with mortality in the multivariate analysis that includes depression, age, gender and serum albumin levels (Supplemental Table S2). The result suggests confounding effects among these items. For example, depression reflects the psychological domain, and the albumin levels or the nutritional status anchor many items in measuring quality of life. The interrelationship between age and these three factors is also complex. Literature and our data have shown that older hemodialysis patients may suffer worse quality of sleep^22^ and quality of life^23^, but age is not associated with depression^14^ (Supplemental Table S6). This is a reason why quality of sleep is no longer associated with mortality but depression remains so when age is factored in. However, quality of life remains associated with mortality when age is considered, and this is compatible with the notion that there are components independent of age within quality of life that are important to mortality.

We used many analytic methods to identify the questions in the quality of life questionnaire that are answered differently between survivors and non-survivors, because the methods examine the questions from a variety of mathematical angles. Mann-Whitney U test compares the score ranks. Kolmogorov-Smirnov Z test compares the distribution pattern of the answers. Fisher’s exact test is to examine the independence of the answers, and is used for cases of small cell counts. Most of the questions in measuring quality of life are unable to distinguish survivors versus non-survivors (Supplemental Table S4), and this may partially explain why total QOL score failed to make prediction in multivariate analysis (Supplemental Table S2).

It is indeed to our surprise that single questions 10, 14 and 15 may predict mortality independently of depression, age, gender, education and other biochemical test results. It implies that there must be a core element that may be extracted from these three questions. Questions 10 “Do you have enough energy for everyday life?” and 15 “How well are you able to get around?” clearly reflect physical strength. The original intention of question 14 “To what extent do you have the opportunity for leisure activities?” is to evaluate how well the respondent can have an access to leisure activities, which is an environment issue. It is very likely, however, that the respondent may interpret this question as a measurement of his or her physical capability to enjoy leisure activities. Previous studies also showed that the degree of mobility^24^, frequency of physical activity^25^ and ability to perform activities of daily life^26^ in hemodialysis patients are associated with mortality although it is not clear whether they are the most prominent one as we have shown in this study.

The clinical implication of the analysis regarding the subjective perception of quality of life is enormous. To prolong survival, an effort to enhance mobility may be beneficial as inability to move is associated with higher mortality. However, mobility appeared not in good correlation with subjective perception of quality of life. Therefore, to help patients “feel” good quality of life, mobility is unlikely a favorable target. Instead, one should focus on things such as environmental safety, feeling of being respected (represented by questions 8 and 27 to which all statistical tests indicated the answers were the same as that to question 1), acceptance of bodily appearance, information availability, capacity of performing daily activity and satisfaction in sex life (represented by questions 11, 13, 17 and 21 to which all tests except one indicated the answers were the same as that to question 1) (Table 5). Interestingly, only question 17 is in the physical health domain, highlighting the importance of psycho-socio-environmental factors in determining the perception of quality of life.

There are limitations in the study. First, the sample size is relatively small, which may decrease the detection power of more stringent tests such as Kolmogorov-Smirnov Z test and multivariate analysis in Cox proportional hazard regression modeling. For example, when questions 15, 10 and 14 are all in the model (Table 4), the effect of question 15 becomes marginal. We believe that the effect is indeed significant but shows otherwise due to type II error, or false negative result, which is more commonly seen in tests with low sample number. Despite the downside, very powerful association factors remain to be possibly identified in small studies. Second, given the large number of tests, the false positive rates would increase without an adjustment of the alpha level, such as Bonferroni adjustment, which is to set the level of significance as alpha divided by testing times. In Table 1, Tables S1, S4 and S5, we performed the tests 27, 29, 28 and 28 times, in order to screen predictive factors. In Table 5, the same tests were performed 27 times to identify which questions were answered in a similar, not different, way to question 1 in the WHOQOL-BREF questionnaire. In the first case, it was not necessary to be stringent. In the second case, it was the insignificant results that matter. Therefore, we did not apply an adjustment in this study. Third, evaluation of physical function and many other variables is based on self-reported results. It is ideal to quantify physical function, for instance, by grasp strength or walking distance yet it is much more labor intensive. The fourth concern is selection bias. Those who chose not, were unable or were excluded to join might have impacts on the results. For instance, if weak or drowsy subjects were included, the power of quality of sleep to predict mortality might have been higher. However, the result that inability to move is associated with lower survival would still hold as those subjects were likely less mobile and also less viable.

In conclusion, quality of life is better than depression and quality of sleep to predict long term mortality in patients on chronic hemodialysis. Mobility may serve as a single independent factor for this predictability. Subjective perception of quality of life is more related to psycho-socio-environmental aspects. Whether intervention to improve mobility may prolong life is worth further investigation.

## Methods

We conducted a prospective cohort study. Prior to the study the ethics committee of the Institutional Research Board in the National Taiwan University Hospital Hsin-Chu Branch approved the proposal. All methods were carried out in accordance to relevant guidelines and regulations. We obtained informed consent from all subjects. The subjects were recruited from a hemodialysis center in a secondary hospital during September 2012 to September 2013. It was a government-run, secondary 819-bed hospital located in an urban area of northern Taiwan. There had been around 250 individuals receiving hemodialysis regularly from whom the study subjects were drawn. The inclusion criteria were (1) at least 6 months of hemodialysis treatment, (2) age older than or equal to 20 years, and (3) in a mental status being capable of answering the questionnaires. We excluded those with advanced cancer (stage 4) and those with tracheostomy. There were 193 patients eligible, from whom 151 signed the informed consent. Those who did not join mostly cited privacy as the reason.

Quality of life was assessed by the World Health Organization Quality of Life assessment questionnaire, an abbreviated version (WHOQOL-BREF). It is composed of 28 questions that cover four major functions, or called domains, namely physical health, psychological health, social relationships and environment. Higher score indicates better quality of life. We used Taiwanese Depression Questionnaire (TDQ) to assess depression by asking subjects the frequency of 18 conditions in the past one week. The frequency scale ranges from 0 (never or seldom), 1 (sometimes), 2 (often) to 3 (very often or always). Total score equal to or higher than 19 is regarded as depression, with sensitivity 89% and specificity 92%^27^. Quality of sleep was measured by the Athens Insomnia Scale (AIS), which consists of 8 questions, among which first five ones are related to nocturnal sleep and last three to daytime dysfunction. Each is rated by 0–3 scale, and the total score higher than or equal to 6 is regarded as poor quality of sleep^28^. AIS has been validated in cancer patients in Taiwan^29^. Three registered nurses interviewed the subjects face-to-face to fill the questionnaires, and collected their demographic, biochemistry and hemodialysis-related data, including their marital status, education, occupation, religion, monthly income and residential status. The lowest cut point of monthly income at 300 US dollars (approximately 10,000 New Taiwan Dollars) was about the value per individual per month that the municipal governments in this country set to define a low income family. Another investigator followed this cohort for more than three years for mortality.

Statistics used to test the differences were as indicated in the results. We applied the Box’s M Test of Equality of Covariance Matrices and calculated the Wilks’ Lambda value to determine whether the data set was suitable for linear discriminant analysis, which was used to compare the discriminating powers among factors for the survival status. To make odds ratios comparable among logistic regression models, the scales of TDQ and AIS were transformed to span from 0 to 112, the same range as the quality of life scale. Also for comparability, the scale of the WHOQOL-BREF was set in reverse order so that higher score means worse condition, the same trend as depression and quality of sleep. Positive predictive value of death was calculated from the data in the classification table with cut value at 0.5. To compare area under curve (AUC), we calculated (AUC1 − AUC2)^2^/(s1^2^ + s2^2^), which fits a Chi square distribution with degree of freedom equal to one (s stands for standard deviation). Weights of evidence (WOE) was calculated using the formula WOE = natural logarithm (% of survival/% of death). Information value was sum of (% of survival/% of death) times WOE. In Table 5 and Supplemental Table S4, the answer responses were treated as categorical variables in Mann-Whitney U test and Kolmogorov-Smirnov Z test. We used SPSS 20 to perform the statistical analyses, except the comparison between area-under-curve values. *p* value lower than 0.05 was considered significant.

## Electronic supplementary material

Supplemental Figures and Tables

## References

[1] Kendrick T (2016). Routine use of patient reported outcome measures (PROMs) for improving treatment of common mental health disorders in adults. The Cochrane database of systematic reviews.

[2] Ghislain I (2016). Health-related quality of life in locally advanced and metastatic breast cancer: methodological and clinical issues in randomised controlled trials. The Lancet. Oncology.

[3] Chang S (2011). Health span or life span: the role of patient-reported outcomes in informing health policy. Health policy.

[4] Xie G (2014). Baseline health-related quality of life and 10-year all-cause mortality among 1739 Chinese adults. PloS one.

[5] Idler EL, Benyamini Y (1997). Self-rated health and mortality: a review of twenty-seven community studies. Journal of health and social behavior.

[6] Diouf M (2014). Could baseline health-related quality of life (QoL) predict overall survival in metastatic colorectal cancer? The results of the GERCOR OPTIMOX 1 study. Health and quality of life outcomes.

[7] Lopes AA (2002). Depression as a predictor of mortality and hospitalization among hemodialysis patients in the United States and Europe. Kidney international.

[8] Schron E, Friedmann E, Thomas SA (2014). Does health-related quality of life predict hospitalization or mortality in patients with atrial fibrillation?. Journal of cardiovascular electrophysiology.

[9] Mathai SC (2016). Health-related Quality of Life and Survival in Pulmonary Arterial Hypertension. Annals of the American Thoracic Society.

[10] Porter AC (2016). Predictors and Outcomes of Health-Related Quality of Life in Adults with CKD. Clinical journal of the American Society of Nephrology: CJASN.

[11] Liebman S, Li NC, Lacson E (2016). Change in quality of life and one-year mortality risk in maintenance dialysis patients. Quality of life research: an international journal of quality of life aspects of treatment, care and rehabilitation.

[12] Perl, J. *et al*. Association between changes in quality of life and mortality in hemodialysis patients: results from the DOPPS. Nephrology, dialysis, transplantation: official publication of the European Dialysis and Transplant Association - European Renal Association, 10.1093/ndt/gfw233 (2016). 10.1093/ndt/gfw233 PMC5837512 27270292

[13] Preljevic VT (2013). Anxiety and depressive disorders in dialysis patients: association to health-related quality of life and mortality. General hospital psychiatry.

[14] Araujo SM (2012). Risk factors for depressive symptoms in a large population on chronic hemodialysis. International urology and nephrology.

[15] Scalo J, Desai P, Rascati K (2015). Insomnia, hypnotic use, and health-related quality of life in a nationally representative sample. Quality of life research: an international journal of quality of life aspects of treatment, care and rehabilitation.

[16] Iliescu EA (2003). Quality of sleep and health-related quality of life in haemodialysis patients. Nephrology, dialysis, transplantation: official publication of the European Dialysis and Transplant Association - European Renal Association.

[17] Chen CK (2010). Depression and suicide risk in hemodialysis patients with chronic renal failure. Psychosomatics.

[18] Brekke FB (2014). Self-perceived quality of sleep and mortality in Norwegian dialysis patients. Hemodialysis international. International Symposium on Home Hemodialysis.

[19] Osthus TB (2012). Mortality and health-related quality of life in prevalent dialysis patients: Comparison between 12-items and 36-items short-form health survey. Health and quality of life outcomes.

[20] Knight EL, Ofsthun N, Teng M, Lazarus JM, Curhan GC (2003). The association between mental health, physical function, and hemodialysis mortality. Kidney international.

[21] Zuluaga MC (2010). Generic and disease-specific quality of life as a predictor of long-term mortality in heart failure. European journal of heart failure.

[22] Chen WC (2006). Sleep behavior disorders in a large cohort of chinese (Taiwanese) patients maintained by long-term hemodialysis. American journal of kidney diseases: the official journal of the National Kidney Foundation.

[23] Abdel-Kader K (2009). Individual quality of life in chronic kidney disease: influence of age and dialysis modality. Clinical journal of the American Society of Nephrology: CJASN.

[24] Sexton DJ (2016). Do patient-reported measures of symptoms and health status predict mortality in hemodialysis? An assessment of POS-S Renal and EQ-5D. Hemodialysis international. International Symposium on Home Hemodialysis.

[25] Lopes AA (2014). Associations of self-reported physical activity types and levels with quality of life, depression symptoms, and mortality in hemodialysis patients: the DOPPS. Clinical journal of the American Society of Nephrology: CJASN.

[26] Jassal SV (2016). Functional Dependence and Mortality in the International Dialysis Outcomes and Practice Patterns Study (DOPPS). American journal of kidney diseases: the official journal of the National Kidney Foundation.

[27] Lee Y, Yang MJ, Lai TJ, Chiu NM, Chau TT (2000). Development of the Taiwanese Depression Questionnaire. Chang Gung medical journal.

[28] Soldatos CR, Dikeos DG, Paparrigopoulos TJ (2000). Athens Insomnia Scale: validation of an instrument based on ICD-10 criteria. Journal of psychosomatic research.

[29] Sun JL, Chiou JF, Lin CC (2011). Validation of the Taiwanese version of the Athens Insomnia Scale and assessment of insomnia in Taiwanese cancer patients. Journal of pain and symptom management.

