# Supplementary material for: A single question regarding mobility in the World Health Organization quality of life questionnaire predicts 3-year mortality in patients receiving chronic hemodialysis
Source: Sci Rep. 2017 Sep 20;7:11981. doi: 10.1038/s41598-017-12276-9 (PMC5607280; doi:10.1038/s41598-017-12276-9)
Supplement: Supplementary file 1 — Supplemental Figures and Tables [file 41598_2017_12276_MOESM1_ESM.pdf]

A single question regarding mobility in the World Health Organization quality of life questionnaire predicts 3-year mortality in patients receiving chronic hemodialysis

Hsiu-HoWang, Miao-Chun Ho, Kuan-Yu Hung, Hui-Teng Cheng

Supplemental Figure S1

Kaplan-Meier survival analysis based on subgroups of quality of life, depression and quality of sleep.

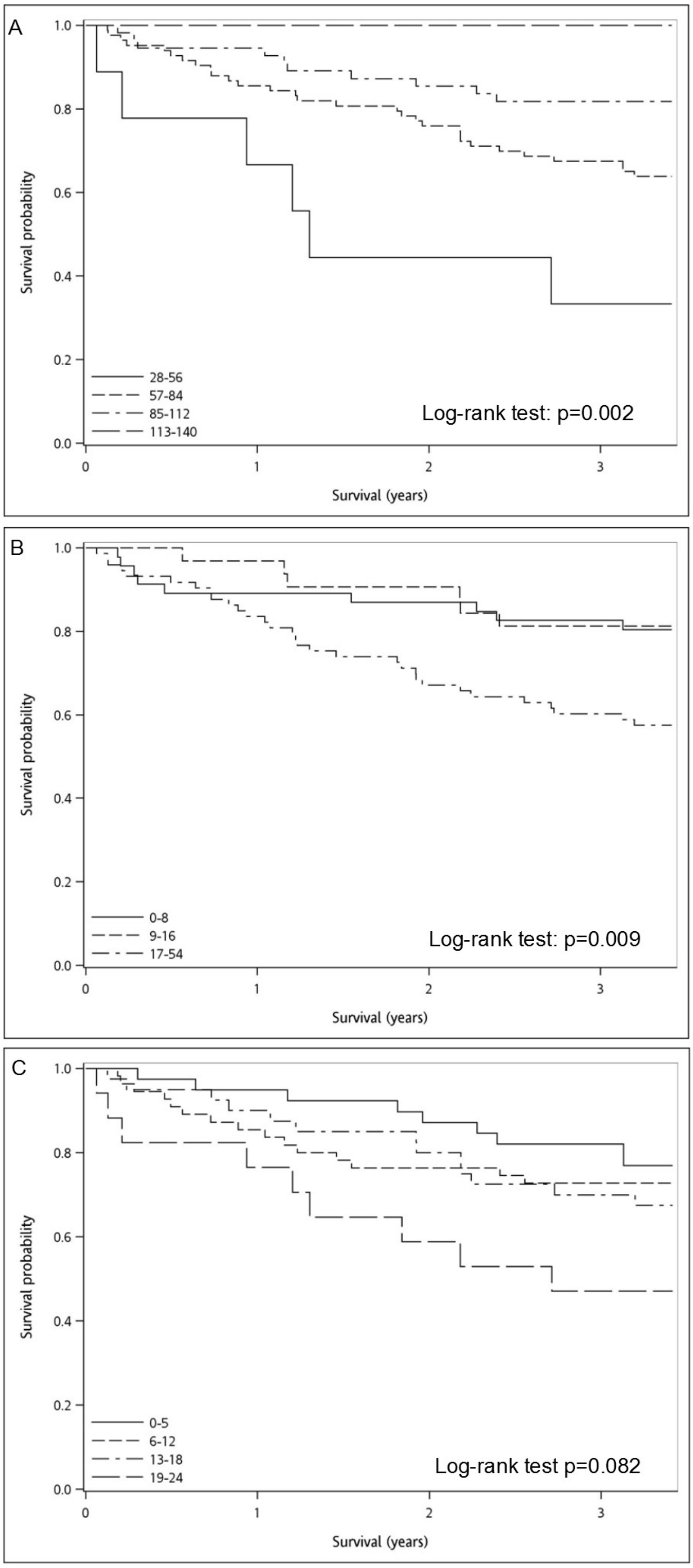

Supplemental Figure S2

Receiver Operating Characteristic curve (sensitivity versus 1-specificity) of quality of life, depression and quality of sleep in relation to mortality. Values of area under curve (AUC), standard deviation and 95% confidence interval are labeled.

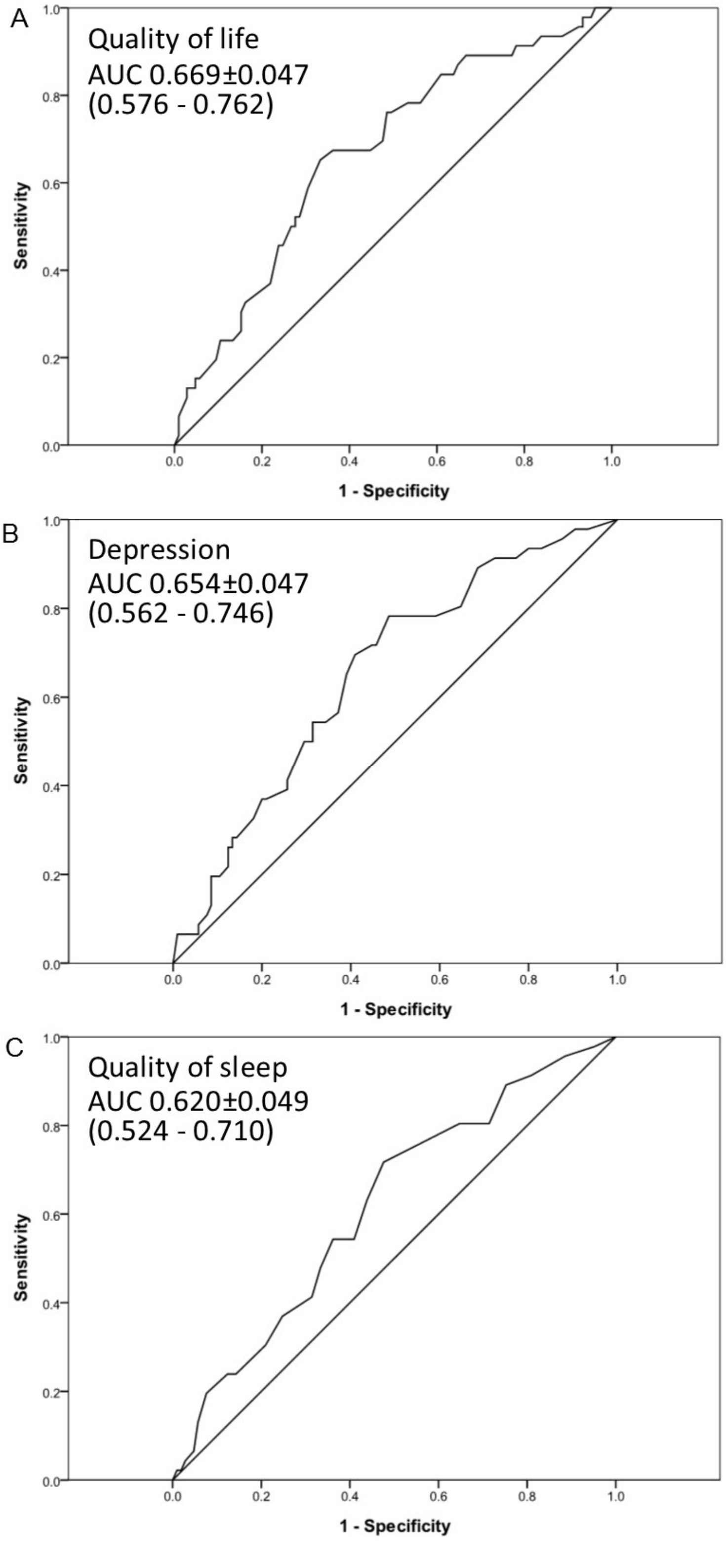

Supplemental Figure S3

Information value plots of quality of life, depression and quality of sleep, of four domains and of 28 individual questions in the WHOQOL-BREF questionnaire.

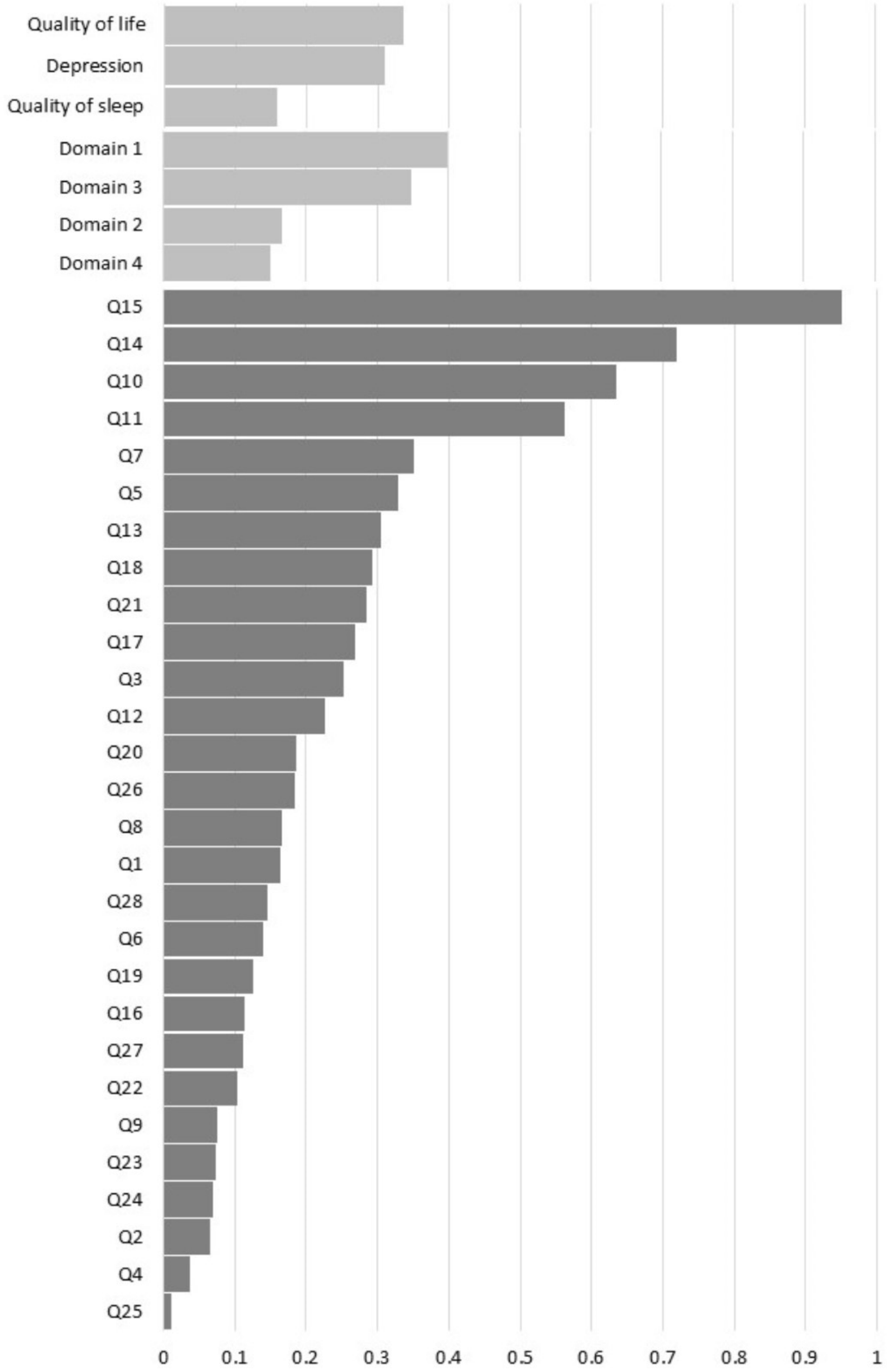

Supplemental Table S1. Hazard ratio of mortality in association with demographic and biochemistry variables by Cox proportional hazard regression model. Data are shown as hazard ratio, calculated by exponential of beta (Exp(B)), with 95% confidence interval.

variables	HR	95% CI	p value
Quality of life	0.966	0.947 - 0.985	**0.000**
Depression TDQ	1.027	1.008 - 1.046	**0.005**
Quality of sleep	1.059	1.010 - 1.110	**0.017**
Age	1.036	1.012 - 1.060	**0.003**
Gender			
men	ref		
women	1.853	1.018 - 3.374	**0.044**
Marital status			
married	ref		
single	0.276	0.038 -2.014	0.204
widowed	1.249	0.556 - 2.807	0.591
divorced	2.510	0.604 - 10.438	0.206
Education			
no	ref		
< 6years	1.179	0.519 - 2.678	0.694
6-9 years	0.286	0.074 - 1.106	0.070
9-12 years	0.175	0.045 - 0.678	**0.012**
college	0.378	0.079 - 1.822	0.226
Occupation			
unemployed	ref		
employed	0.273	0.066 - 1.128	0.073
Religion			
nil	ref		
Buddhism	0.704	0.206 - 2.403	0.576
Christianity	1.610	0.737 - 3.517	0.232
Catholicism	0.687	0.347 - 1.360	0.282
Taoism	0.000	0.000	.
miscellaneous	0.000	0.000	.
Monthly income (USD)			
<300	ref		
300-1000	0.776	0.391 - 1.539	0.468
1000-2000	0.129	0.018 - 0.946	**0.044**
>2000	0.451	0.108 - 1.882	0.275

Living			
alone	ref		
with family	0.549	0.133 - 2.272	0.408
other	0.996	0.182 - 5.445	0.997
BUN	0.992	0.979 - 1.006	0.255
Creatinine	0.856	0.766 - 0.958	**0.007**
Sodium	0.964	0.902 - 1.031	0.286
Potassium	1.061	0.781 - 1.441	0.704
Calcium	0.777	0.548 - 1.103	0.158
Phosphate	0.882	0.722 - 1.079	0.223
Ca x P product	0.984	0.964 - 1.004	0.116
Albumin	0.334	0.149 - 0.748	**0.008**
Glucose	1.004	1.000 - 1.008	**0.050**
AST	1.006	0.981 - 1.033	0.630
ALT	0.996	0.979 - 1.014	0.670
Kt/V of urea	1.356	0.352 - 5.226	0.659
Urea reduction rate (URR)	2.898	0.021 - 393.978	0.671
Dialysis vintage	1.053	0.983 - 1.127	0.141
Alkaline phosphatase	1.002	1.000 - 1.004	**0.045**
Triglyceride	0.999	0.997 - 1.002	0.581
Cholesterol	0.997	0.989 - 1.006	0.511
Ferritin	1.000	1.000 - 1.001	0.346
i-parathyroid hormone	1.000	0.999 - 1.001	0.558
Hemoglobin	0.967	0.800 - 1.169	0.728
Hematocrit	0.999	0.934 - 1.069	0.974
MCV	1.015	0.983 - 1.048	0.358
Platelet	1.004	1.000 - 1.008	0.054

ALKP: alkaline phosphatase, ALT: Alanine aminotransferase, AST: Aspartate aminotransferase, BUN: blood urea nitrogen, Ca x P: calcium and phosphate product, i-parathyroid hormone: intact parathyroid hormone, Kt/V: dialysis adequacy by dialyzer clearance of urea (K) times time (t) divided by volume (V) of distribution of urea, MCV: mean corpuscular volume of red blood cell, QOL: quality of life, QOS: quality of sleep, USD: US dollar.

Supplemental Table S2. Cox proportional hazard regression analysis for mortality by multiple variables. Data are shown as hazard ratio, or exponential of beta (Exp(B)), of mortality, with 95% confidence interval in parenthesis. na: non-applicable

	Models with two or three factors in				Models with one or two factors in, plus age, gender and albumin				Models with indicated factors plus education, alkaline phosphatase and glucose		
QOL	**0.967 (0.939-0.997)**	**0.966 (0.942-0.991)**		**0.967 (0.938-0.998)**	**0.975 (0.954-0.996)**	na	na	1.003 (0.947-1.009)	**0.972 (0.946-0.998)**	0.975 (0.950-1.001)	0.976 (0.944-1.009)
Depression	1.002 (0.973-1.033)		1.021 (0.996-1.047)	1.002 (0.970-1.035)	na	**1.020 (1.000-1.041)**	na	0.977 (0.973-1.035)	na	na	1.000 (0.968-1.003)
Sleep		1.003 (0.943-1.066)	1.021 (0.961-1.047)	1.001 (0.937-1.069)	na	na	1.034 (0.982-1.088)	na	0.995 (0.934-1.059)	0.997 (0.936-1.063)	na
Age					**1.026 (1.002 -1.050)**	**1.029 (1.005-1.053)**	**1.027 (1.003-1.051)**	**1.026 (1.003-1.051)**	1.006 (0.979-1.035)	1.006 (0.978-1.035)	1.006 (0.977-1.036)
Gender (women)					1.329 (0.715-2.473)	1.355 (0.732-2.508)	1.315 (0.695-2.486)	1.324 (0.712-2.464)	1.234 (0.601-2.534)	1.258 (0.627-2.521)	1.247 (0.633-2.458)
Albumin					0.585 (0.240-1.428)	0.516 (0.211-1.259)	0.451 (0.168-1.093)	0.587 (0.240-1.437)	na	0.495 (0.206-1.192)	0.494 (0.205-1.189)
Creatinine					na	na	na	na	0.996 (0.833-1.119)	na	na

Supplemental Table S3. Logistic regression analysis for odds ratio of mortality in relation to quality of life, depression or quality of sleep. In upper panel all three scores are transformed into the same scale (28-140 or 0-112). Odds ratio is calculated by exponential of beta (Exp(B)). Positive predictive value of death is calculated from the data in the classification table with cut value set at 0.5.

	Odds ratio (Exp (B))	95% confidence interval		p value	Positive predictive value of death (predicted/observed)
Quality of life (reverse order)	1.040	1.015	1.065	**0.001**	66.7% (6/9)
Depression (0-112)	1.017	1.005	1.029	**0.005**	40.0% (4/10)
Quality of Sleep (0-112)	1.015	1.002	1.027	**0.024**	50.0% (1/2)
Quality of life (28-140)	0.961	0.939	0.985	**0.001**	
Depression (0-54)	1.035	1.010	1.061	**0.005**	
Quality of Sleep (0-24)	1.070	1.009	1.134	**0.024**	

Supplemental Table S4. Comparisons in each of 28 individual questions in the quality of life questionnaire (WHOQOL-BREF) between survivors and non-survivors.

	Questions	Mann-Whitney U test (p value)	Kolmogorov-Smirnov Z test (p value)	Chi square and p values		Fisher exact test and p value	
General	(Q1) How would you rate your quality of life?	**0.034**	0.573	4.991	0.292	5.136	0.259
	(Q2) How satisfied are you with your health?	0.208	0.841	1.966	0.596	1.815	0.626
Domain 1(physical health)	(Q3) To what extent do you feel that physical pain prevents you from doing what you need to do?	**0.008**	0.123	7.909	0.095	7.654	0.101
	(Q4) How much do you need any medical treatment to function in your daily life?	0.617	0.999	1.132	0.887	1.141	0.903
	(Q10) Do you have enough energy for everyday life?	**0.001**	**0.001**	**10.743**	**0.000**	**20.695**	**0.000**
	(Q15) How well are you able to get around?	**0.000**	**0.000**	**28.445**	**0.000**	**27.630**	**0.000**
	(Q16) How satisfied are you with your sleep?	0.486	0.831	3.682	0.454	3.623	0.500
	(Q17) How satisfied are you with your ability to perform your daily living activities?	**0.004**	0.061	9.214	0.054	8.444	0.064
	(Q18) How satisfied are you with your capacity for work?	**0.004**	**0.020**	**9.838**	**0.038**	**9.242**	**0.043**
Domain 2 (psychological)	(Q5) How much do you enjoy life?	**0.017**	0.115	**10.714**	**0.027**	**10.425**	**0.026**
	(Q6) To what extent do you feel your life to be meaningful?	0.148	0.511	4.567	0.343	4.577	0.325
	(Q7) How well are you able to concentrate?	**0.011**	0.169	**12.094**	**0.006**	**11.182**	**0.010**
	(Q11) Are you able to accept your bodily appearance?	**0.044**	**0.020**	**17.030**	**0.002**	**16.907**	**0.001**
	(Q19) How satisfied are you with yourself?	0.093	0.347	3.937	0.432	3.707	0.454
	(Q26) How often do you have negative feelings such as blue mood, despair, anxiety, depression?	0.061	0.350	5.105	0.282	5.142	0.271
Domain 3 (social)	(Q20) How satisfied are you with your personal relationships?	**0.019**	0.293	5.791	0.213	5.999	0.169
	(Q21) How satisfied are you with your sex life?	**0.029**	0.151	**10.153**	**0.029**	**9.401**	**0.036**
	(Q22) How satisfied are you with the support you get from your friends?	0.109	0.938	8.810	0.055	7.465	0.080
	(Q27) Do you feel you are respected?	0.114	0.819	4.241	0.377	3.744	0.425
(environment	(Q8) How safe do you feel in your daily life?	0.109	0.463	5.734	0.222	5.552	0.219
	(Q9) How healthy is your physical environment?	0.347	0.970	6.118	0.192	5.510	0.214
	(Q12) Have you enough money to meet your needs?	0.789	0.960	6.279	0.180	6.461	0.158

(Q13) How available to you is the information that you need in your day-to-day life?	0.077	0.386	**10.603**	**0.029**	**9.524**	**0.040**
(Q14) To what extent do you have the opportunity for leisure activities?	**0.000**	**0.001**	**17.689**	**0.001**	**18.080**	**0.001**
(Q23) How satisfied are you with the conditions of your living place?	0.529	0.999	6.020	0.195	6.056	0.166
(Q24) How satisfied are you with your access to health services?	0.856	1.000	1.931	0.793	1.868	0.828
(Q25) How satisfied are you with your transport?	0.672	0.999	3.731	0.455	3.963	0.399
(Q28) Can you usually eat what you want to eat?	0.091	0.292	4.511	0.351	4.814	0.295

Supplemental Table S5. Hazard ratio of mortality in association with each of 28 individual questions in the quality of life questionnaire (WHOQOL-BREF) by Cox proportional hazard regression model. Data are shown as hazard ratio, calculated by exponential of beta (Exp(B)), with 95% confidence interval. Model includes TDQ depression score, age, gender, education, albumin, glucose and alkaline phosphatase.

		HR	95% CI		p value
General	Q1	0.873	0.587	1.300	0.505
	Q2	1.182	0.818	1.708	0.374
Domain 1 (physical health)	Q3	0.909	0.707	1.169	0.456
	Q4	1.207	0.949	1.536	0.125
	Q10	**0.577**	**0.384**	**0.867**	**0.008**
	Q15	**0.598**	**0.431**	**0.829**	**0.002**
	Q16	1.483	0.998	2.203	0.051
	Q17	0.766	0.529	1.109	0.157
	Q18	0.822	0.585	1.154	0.257
Domain 2 (psychological)	Q5	0.759	0.537	1.071	0.117
	Q6	1.024	0.713	1.471	0.896
	Q7	0.779	0.501	1.210	0.266
	Q11	0.780	0.549	1.108	0.166
	Q19	1.000	0.737	1.356	1.000
	Q26	1.050	0.753	1.462	0.775
Domain 3 (social)	Q20	0.661	0.401	1.090	0.105
	Q21	0.704	0.466	1.061	0.094
	Q22	0.859	0.568	1.300	0.473
	Q27	0.712	0.486	1.044	0.082
Domain 4 (environmental)	Q8	0.818	0.551	1.216	0.321
	Q9	0.898	0.589	1.371	0.619
	Q12	0.959	0.728	1.264	0.766
	Q13	0.851	0.605	1.198	0.357
	Q14	**0.676**	**0.481**	**0.950**	**0.024**
	Q23	1.048	0.679	1.617	0.832
	Q24	1.315	0.849	2.035	0.219
	Q25	1.060	0.691	1.626	0.790
	Q28	0.984	0.699	1.387	0.928

Supplemental Table S6. Pearson correlation coefficient between age and each of three scores.

		age	Quality of life score	TDQ score (Depression)	Quality of life score
age	Pearson correlation	1	**0.204**	0.137	**-0.242**
	p value (two-tailed)		**0.012**	0.095	**0.003**
Quality of life score	Pearson correlation	0.204	1	**0.703**	**-0.671**
	p value (two-tailed)	0.012		**0.000**	**0.000**
TDQ score (Depression)	Pearson correlation	0.137	0.703	1	**-.755**
	p value (two-tailed)	0.095	0.000		**0.000**
Quality of life score	Pearson correlation	-0.242	-0.671	-0.755	1
	p value (two-tailed)	0.003	0.000	0.000	
